# Non-Transfusion-Dependent Thalassemia: A Complex Mix of Genetic Entities Yet to Be Fully Discovered

**DOI:** 10.1155/2015/161434

**Published:** 2015-04-06

**Authors:** Paolo Ricchi, Aldo Filosa, Aurelio Maggio, Suthat Fucharoen

**Affiliations:** ^1^U.O.S.D. Centro delle Microcitemie “A. Mastrobuoni”, AORN A. Cardarelli, 80131 Naples, Italy; ^2^U.O.C. Ematologia II, Ospedali Riuniti Villa Sofia-Cervello, 90146 Palermo, Italy; ^3^Thalassemia Research Center, Institute of Molecular Biosciences, Mahidol University, Nakhon Pathom 73170, Thailand

The management of patients with non-transfusion-dependent thalassaemia (NTDT) has been a challenging task: in fact, within this conventional definition, clinicians have to deal with a great variety of syndromes mixed in terms of their molecular background, clinical course, and severity which share only the characteristics that are not entirely dependent on transfusions [1, 2]. In fact, NTDT phenotypes include patients with *β*-thalassemia intermedia, hemoglobin E/*β*-thalassemia, and Hemoglobin H disease (*α*-thalassemia intermedia) but also those with structural variant of hemoglobin associated with “*α*” or “*β*” thalassemia in heterozygous condition which often have analogous characteristics [3]. However, among different genetic entities of NTDT, the lack of a clear genotype-phenotype relationship further complicates this complex and extensive scenario in clinical practice [4]. Thus, despite the availability of recent guideline from Thalassemia International Federation, the strength of several treatments and follow-up recommended strategies should be confirmed in selected population [5]; in fact, most of these recommendations arise from retrospective and cross sectional studies where different patients were heterogeneously treated along their life with occasional transfusions, iron chelation, and splenectomy. Furthermore, in clinical practice, most of these recommendations are difficult to transfer into the “wide-spectrum” of phenotypes particularly in the case of patients first diagnosed in adult life.

This special issue was launched to provide some insights into this wide subject, which is continually evolving. Out of the five articles published in this special issue on NTDT, two are updated reviews. It is well worth it to note that one focused on the role of oxidative damage by reactive oxygen species (generated by free globin chains and labile plasma iron) as a potential additive contributor to cell injury, tissue damage, and hypercoagulability observed in patients with NTDT; the other was devoted to expanding the emerging setting of endocrine and bone disease in NTDT. We collected also three original articles, two describing the profile of HbH disease in Taiwan and its incidence in Lebanon, respectively, and one evaluating again the endocrine and bone disease in *β*-thalassemia intermedia patients. Obviously, some of this epidemiological information could be only of regional interest but certainly adds some more clinical useful information.

In conclusion, despite the great effort and benefit to encompass such a variety of syndromes in the acronyms NTDT, we should be always prepared to face the limitation of mixing heterogeneous patients within a single category. Further studies are needed to better define and compare at clinical and molecular level the different genetic entities of NTDT. The recent finding of similar degrees of anemia but diverse patterns of the GDF15-hepcidin-ferritin axis between *β*-thalassemia intermedia and HbH disease in a Sardinian series should stimulate extending such comparative evaluation among all different classes of NTDT [6]. We hope that readers of this special issue will find our articles accurate and updated and will focus on the need of developing more studies in this intricate and not fully explored setting.

*Paolo Ricchi* *Aldo Filosa* *Aurelio Maggio* *Suthat Fucharoen*
